# Neuropeptides neurotensin and substance P accelerate diabetic wound healing by modulating immunity and the skin microbiome

**DOI:** 10.1038/s41598-025-30723-w

**Published:** 2026-02-17

**Authors:** Ana Maranha, Ermelindo C. Leal, Susana Alarico, Igor Tiago, Sónia G. Pereira, Nuno Empadinhas, Eugénia Carvalho

**Affiliations:** 1 CNC - Center for Neuroscience and Cell Biology, University of Coimbra, Coimbra, 3004-504 Portugal; 2 CIBB - Centre for Innovative Biomedicine and Biotechnology, University of Coimbra, Coimbra, 3004-504 Portugal; 3 Department of Life Sciences, University of Coimbra, Coimbra, 3000-456 Portugal; 4 CFE – Centre for Functional Ecology, University of Coimbra, Coimbra, 3000-456 Portugal; 5 ciTechCare - Center for Innovative Care and Health Technology, School of Health Sciences (ESSLei), Polytechnic University of Leiria, Leiria, 2414-016 Portugal

**Keywords:** Neuropeptides, Substance P, Neurotensin, Skin microbiota, Diabetic wound healing, Inflammation, Dysbiosis, Diseases, Immunology, Microbiology

## Abstract

Diabetic foot ulcers (DFUs) are a common and debilitating complication of diabetes mellitus, typified by impaired healing, persistent inflammation, and skin microbiota dysbiosis. Although some neuropeptides are immunomodulatory, their effects on skin microbiota and wound resolution remain largely unexplored. We examined the therapeutic potential of the neuropeptides neurotensin (NT) and substance P (SP) in reshaping inflammatory and microbial dynamics and accelerating wound closure in a healthy and streptozotocin-induced diabetic mice model. Full-thickness cutaneous wounds were induced on both groups, followed by daily topical applications of saline (CT), NT, or SP. Wound closure was monitored and changes in skin bacterial communities characterized using next-generation sequencing. Wound tissue samples were collected for immunohistochemical analysis. Topical application of NT or SP significantly accelerated wound healing, with the most pronounced effects in diabetic animals. Both neuropeptides reduced the number of pro-inflammatory cells, lowering M1 macrophages in diabetic wounds from 16.4 ± 4 to 12.1 ± 3 with NT and 10.9 ± 3 with SP, while increasing M2 macrophages from 11.6 ± 4 to 18.1 ± 4 (NT) and 21.6 ± 5 (SP). SP exerted the most pronounced immunomodulatory effect, normalizing the M1/M2 ratio and reducing the number of CD3⁺ T cells (12.4 ± 3) and neutrophils (12.1 ± 3) to levels comparable to non-diabetic skin. Concurrently, SP treatment induced substantial microbiota remodeling in diabetic wounds, reducing *Staphylococcus* by circa 34%, Aerococcaceae_unclassified by 6.8%, *Aerococcus* by 1.5% and nearly eliminating *Weissella*. These reductions were accompanied by an over twofold increase in commensal genera (*Lactobacillus*, Micrococcaceae_unclassified, Actinobacteria_unclassified), shifting the microbial community toward a more non-diabetic profile. Our findings suggest that NT and especially SP, enhance diabetic wound healing through dual local immunomodulation and microbiota restructuring.

**Supplementary Information:**

The online version contains supplementary material available at 10.1038/s41598-025-30723-w.

## Introduction

Diabetes mellitus (DM) is a major public health problem worldwide, affecting over 536 million people in 2021, and projected to reach 783 million by 2045^1,2^. One of its most debilitating complications is diabetic foot ulceration (DFU), which develops in 19–34% of patients and accounts for significant morbidity, mortality, and healthcare burden^3,4^. DFUs are the leading cause of lower-limb amputations, affecting up to 85% of patients with severe ulcers^5,6^. Their poor healing is driven by sustained inflammation, impaired vascularization, and increased susceptibility to infection. Up to 60% of DFUs progress to diabetic foot infections (DFIs), often requiring hospitalization and long-term antimicrobial use^7^, which contributes to antimicrobial resistance, a problem projected to kill 8.22 million people in 2050^8^.

The skin is the body’s largest protective barrier, hosting diverse microbial communities adapted to its harsh, desiccated, nutrient-limited, and acidic environment. These microorganisms maintain homeostasis by outcompeting pathogens, and producing antimicrobials while also stimulating the production of key host-derived antimicrobial peptides (AMPs), by regulating inflammation and immune responses, and supporting epidermal integrity maintenance^9–12^. Healthy skin is dominated by Actinobacteria (mostly *Cutibacterium* spp., *Corynebacterium* spp. and members of the family Micrococcaceae), Firmicutes (mostly *Staphylococcus* spp.) Proteobacteria, and Bacteroidetes^13^. Disruption of this microbial balance can enhance the susceptibility to skin disorders and infections^14^.

Wound healing is a complex and highly regulated process involving four overlapping phases: hemostasis, inflammation, proliferation, and remodeling^15^. However, in chronic DFUs, this orderly progression is disrupted, often stalling in the inflammatory phase due to a combination of endogenous factors (i.e. underlying pathophysiological conditions such as peripheral vasculopathy, neuropathy, and hyperglycemia) and exogenous factors (i.e. persistent high bacterial burden, biofilm formation, antimicrobial resistance, and evasion of host immune responses). Diabetic wounds exhibit an increased infiltration of pro-inflammatory cells such as neutrophils and macrophages, with persistent activation of pro-inflammatory cytokines including interleukin (IL)-1β, IL-6, and IL-8 and TNF-α^16,17^. Macrophage dysregulation is a key feature in wound healing. Normally, macrophages transition from pro-inflammatory (M1) to pro-repair (M2) states, but in diabetes the imbalance favors prolonged M1 activity^18^. Hyperglycemia further impairs leukocyte clearance of pathogens and apoptotic cells^19,20^. Furthermore, pathogenic bacteria exacerbate this dysregulation by overactivating neutrophils and cytokine production and releasing virulence factors such as the pore forming alpha-toxin or the Epidermal cell differentiation inhibitor (EDIN), that hinder keratinocyte migration, degrade extracellular matrix, and promote biofilm formation^21–23^.

Chronic wounds microbiota often shows reduced diversity and stable, biofilm-driven, communities correlated with delayed healing. These wounds often contain polymicrobial biofilms dominated by *Staphylococcus*, *Pseudomonas*, *Corynebacterium*, and *Streptococcus* species^13,24,25^, with *S. aureus* and *S. epidermidis* being particularly prevalent. Notably, specific strains of *S. aureus*, as well as strict or facultative anaerobes, have been linked to delayed healing and poorer clinical outcomes^24,26^, while some commensals can enhance healing by inducing migration and proliferation of keratinocytes^27^, which highlights the importance of restoring immune and microbial balance in DFUs.

Neuropeptides, endogenous signaling molecules with multifunctional roles in inflammation, tissue regeneration, and host-microbe interactions, emerge as promising therapeutic candidates. Among these, Neurotensin (NT) and Substance P (SP), are key modulators of wound healing processes^28,29^. Both are reduced in diabetic impaired wound healing, SP is diminished in non-healing wounds^29^ while NT is downregulated under hyperglycemia^30^. Both peptides exhibit chemotactic, angiogenic, and immunomodulatory properties improving wound closure in preclinical diabetic models^28–31^. Beyond tissue repair, neuropeptides shape host–microbe communication:^32^, SP and NT modulate gut microbiota composition and function^33,34^ and SP contributes to skin microbial homeostasis^35^. Yet, no studies to date have examined whether neuropeptide-based therapies can restore microbial balance or how such microbial changes interact with immune regulation during wound repair.

Here, we address this gap by investigating the effects of topical SP and NT on both immune dynamics and microbiome remodeling in a diabetic wound model. By integrating macrophage phenotyping, inflammatory cell infiltration, and 16 S rRNA sequencing, we provide a systems-level evaluation of how neuropeptides modulate the wound environment. Our findings reveal that SP and NT not only accelerate healing in diabetic mice but also drive a shift toward a more favorable, non-diabetic–like microbiota while simultaneously promoting resolution of chronic inflammation. This combined neuroimmune and microbiome remodeling identifies a previously uncharacterized mechanism of action and highlights neuropeptide therapy as a promising approach for treating chronic diabetic wounds.

## Results

### Neuropeptides accelerate wound closure in diabetic and non-diabetic mice

**Fig. 1:**
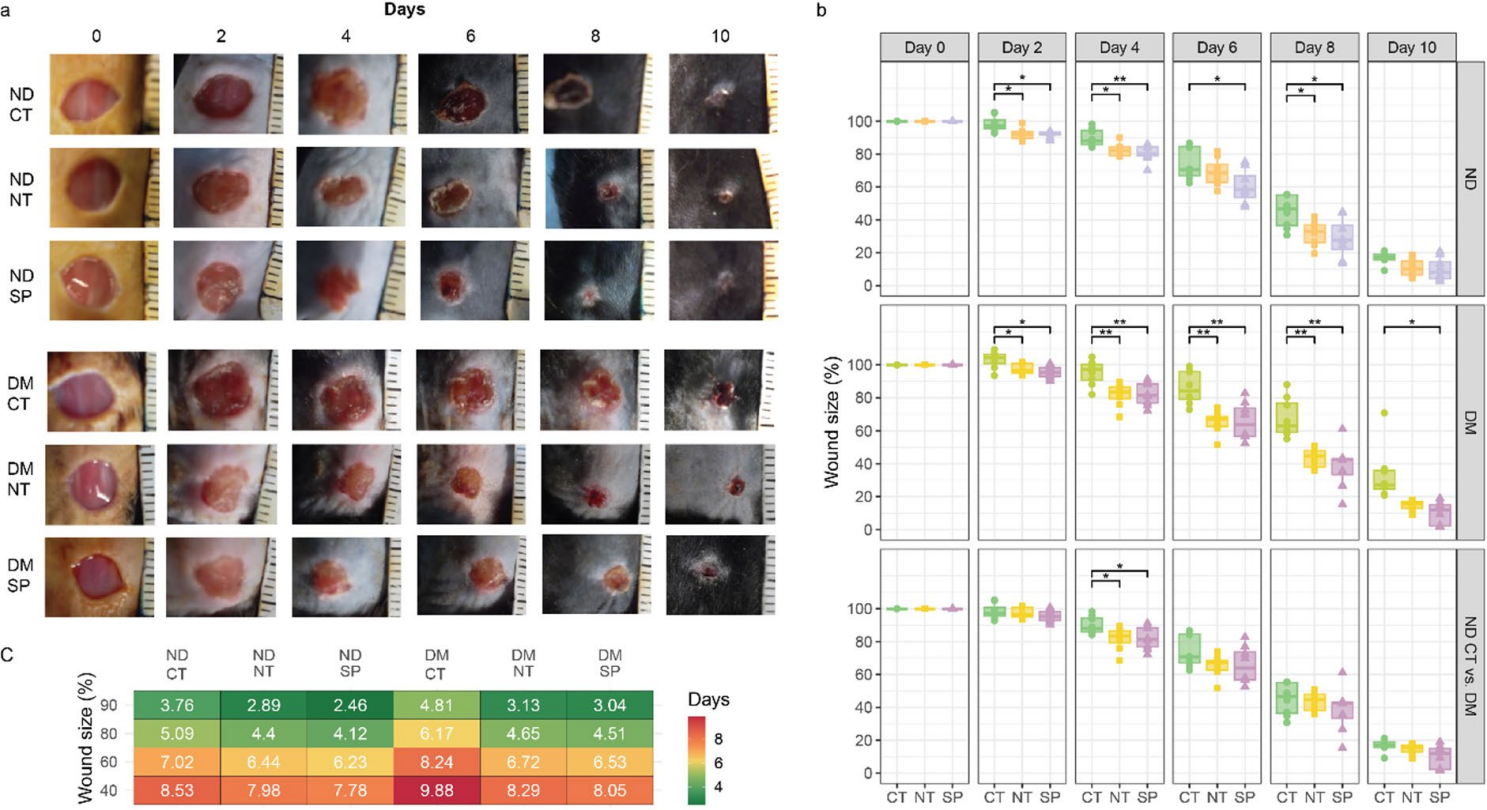
Neuropeptides enhance wound healing in diabetic (DM) and non-diabetic (ND) mice. (**A**) Representative macroscopic images of skin wounds in ND and DM mice treated topically with saline (CT), neurotensin (NT), or substance P (SP), captured from day 0 to day 10 post-wounding. (**B**) Wound area progression over time shown as mean ± SD (*n* = 6 observations per mice per group). (**C**) Time to reach 90%, 80%, 60%, and 40% wound size relative to baseline, estimated via linear regression. *p < 0.05, **p < 0.01, ***p < 0.0001. Test statistics are reported in Table S1.

To assess the effects of the neuropeptides neurotensin (NT) and substance P (SP) on wound healing in vivo, we applied daily each peptide topically to full-thickness excisional wounds in both type 1 diabetes mouse model (DM) and non-diabetic control mice (ND). Diabetic mice exhibited delayed wound healing compared to non-diabetic controls, with a particularly significantly larger wound area observed on day 8 post-wounding (68% ±12% vs. 45% ± 10%, *p* = 0.0034) (Fig. 1A–B, Table S1). Linear regression analysis further confirmed that, overall, wound closure in DM mice lagged behind ND mice by at least one day (Fig. 1C).

NT and SP treatment significantly accelerated wound closure in both ND and DM mice (Fig. 1B–C). In ND mice, NT and SP treatment reduced wound size throughout the experiment by an average of ~ 8% and ~ 11%, respectively, compared to untreated ND mice. These reductions were statistically significant on all measured days, except day 6 for NT and day 10 for both peptides (p-values: NT—day 2: 0.0211, day 4: 0.017, day 8: 0.0174; SP—day 2: 0.0174, day 4: 0.008, day 6: 0.0446, day 8: 0.0174) (Table S1).In DM mice, the effects of NT and SP treatment were even more pronounced. NT treatment reduced wound size by an average of ~ 16%, while SP treatment achieved an average reduction of ~ 19%, compared to saline-treated (CT) DM mice. Remarkably, treated wounds in DM mice were also smaller than those in untreated ND mice by ~ 4% (NT) and ~ 7% (SP) (Fig. 1A, Table S1). These reductions were statistically significant on all measured days, except day 10 for NT (p-values NT: day 2 0.0473, day 4: 0.0081, day 6: 0.0029, day 8: 0.0017; SP: day 2: 0.0174, day 4: 0.0081, day 6: 0.0095, day 8: 0.0017, day 10: 0.0275; Table S2). Moreover, linear regression analysis further revealed that NT and SP treatment accelerated wound healing in DM mice by approximately 1.5 and 1.7 days, respectively, compared to untreated DM mice (Fig. 1C).

Together, these findings suggest that topical administration of NT and SP significantly enhances wound healing in both diabetic and non-diabetic mice. The therapeutic benefit is especially marked in diabetic animals, where healing is typically impaired, with SP showing improved efficacy in accelerating wound closure.

### Neuropeptides exhibit anti-inflammatory properties in diabetic wounds

**Fig. 2:**
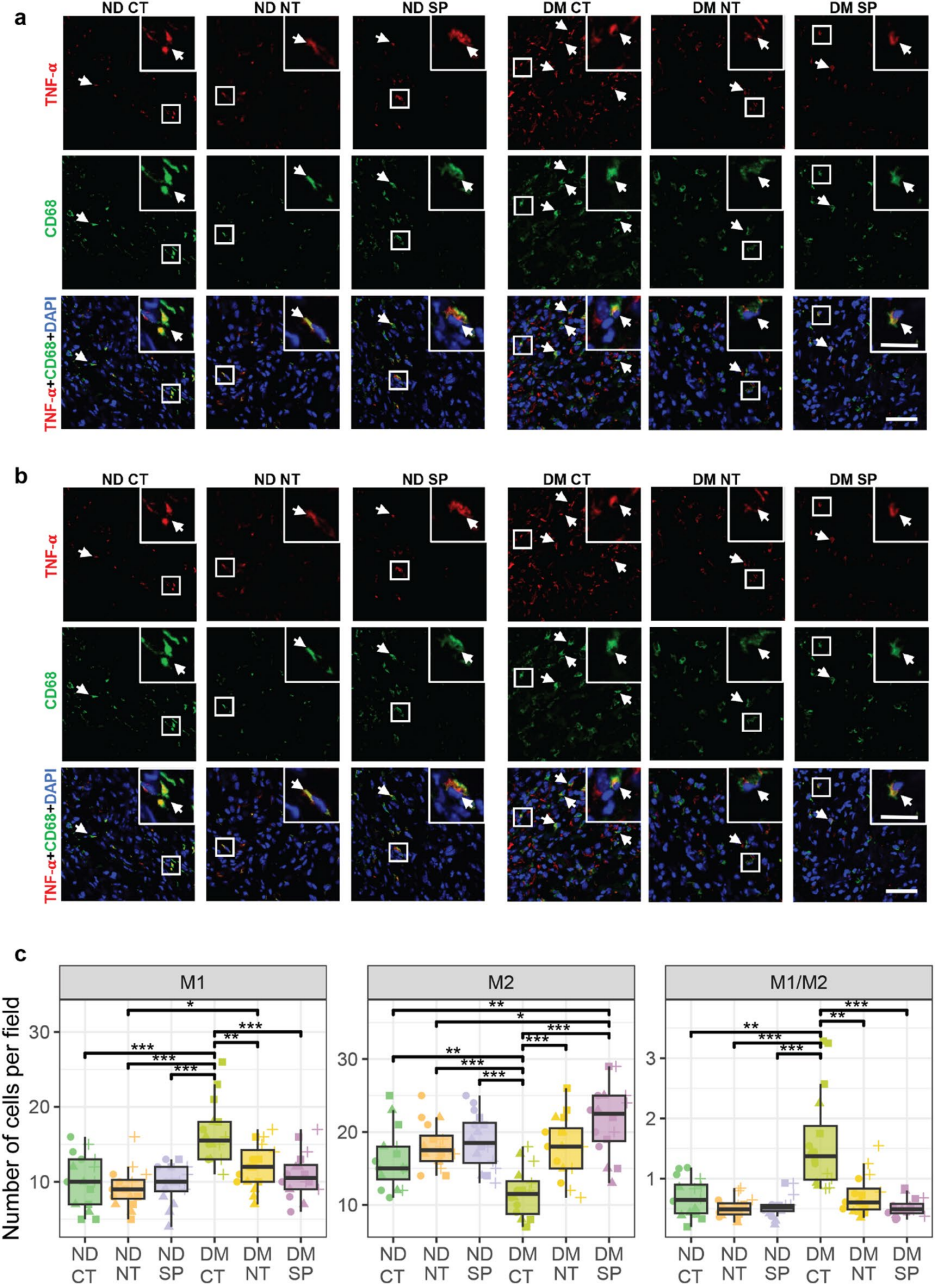
Neuropeptides reduce inflammation in diabetic wounds. (**A**) M1–like macrophage and (**B**) M2-like macrophage. Macrophages were identified by the expression of CD68 (green). M1 and M2-like macrophage phenotypes were determined by TNF-α (red) and CD206 (red), respectively. (**C**) Number of M1- and M2-like macrophages, and M1/M2 ratio per field. The nuclei were stained with DAPI (blue). Scale bar − 50 μm; magnification: scale bar − 20 μm. Data are presented as mean ± SD from 3 biological replicates per group. White arrows identify the staining. Statistical significance was determined using one-way ANOVA with Welch correction and post hoc t-tests. *p < 0.05, **p < 0.01, ***p < 0.0001. Test statistics are reported in Table S2. ND- Non-diabetic; DM- Diabetic; CT – saline; NT-neurotensin; SP-Substance P.

To assess macrophage polarization, we used TNF-α and CD206 as markers for pro-inflammatory (M1) and anti-inflammatory (M2) macrophages, respectively. Quantification of M1 (Fig. 2A, C) and M2 (Fig. 2B, C) macrophage numbers in skin wounds from diabetic mice and from non-diabetic controls as well as the M1/M2 ratio (Fig. 2C), was performed on skin wound biopsies harvested 10 days after wounding.

In ND mice, no significant differences were observed in the number of M1 macrophages across treatment groups. However, CT diabetic wounds exhibited the highest number of M1 macrophages (16.4 ± 4 cells/field), a value significantly greater than all other groups. NT- and SP-treated diabetic wounds showed a marked reduction in M1 macrophages (12.1 ± 3 and 10.9 ± 3, respectively) Notably, M1 macrophage abundance in DM mice SP-treated wounds was not statistically different from that observed in ND mice (Fig. 2A, C, Table S2). Similarly, M2 macrophage numbers did not differ significantly among ND mice, though with slight increases in NT- and SP-treated groups (18.1 ± 3 and 18.8 ± 4, respectively), versus CT (16.2 ± 4). In contrast, CT DM wounds had significantly lower M2 macrophage count (11.6 ± 4), than all other groups. While NT-treated diabetic wounds showed an increase in M2 macrophages (18.1 ± 4), SP-treated diabetic wounds displayed the most pronounced response (21.6 ± 5), a value significantly greater than both ND and DM-NT mice (18.1 ± 3 and 18.1 ± 4, respectively) (Fig. 2B, C, Table S2).

The shift in macrophage phenotype toward a higher M2 presence, particularly in SP-treated wounds, suggests an anti-inflammatory effect that may be beneficial in diabetic wound healing. This was further reflected in the M1/M2 ratio, which was significantly elevated in diabetic wounds but normalized by NT and SP treatment, reaching levels comparable to those in ND mice (Fig. 2C, Table S2).

**Fig. 3:**
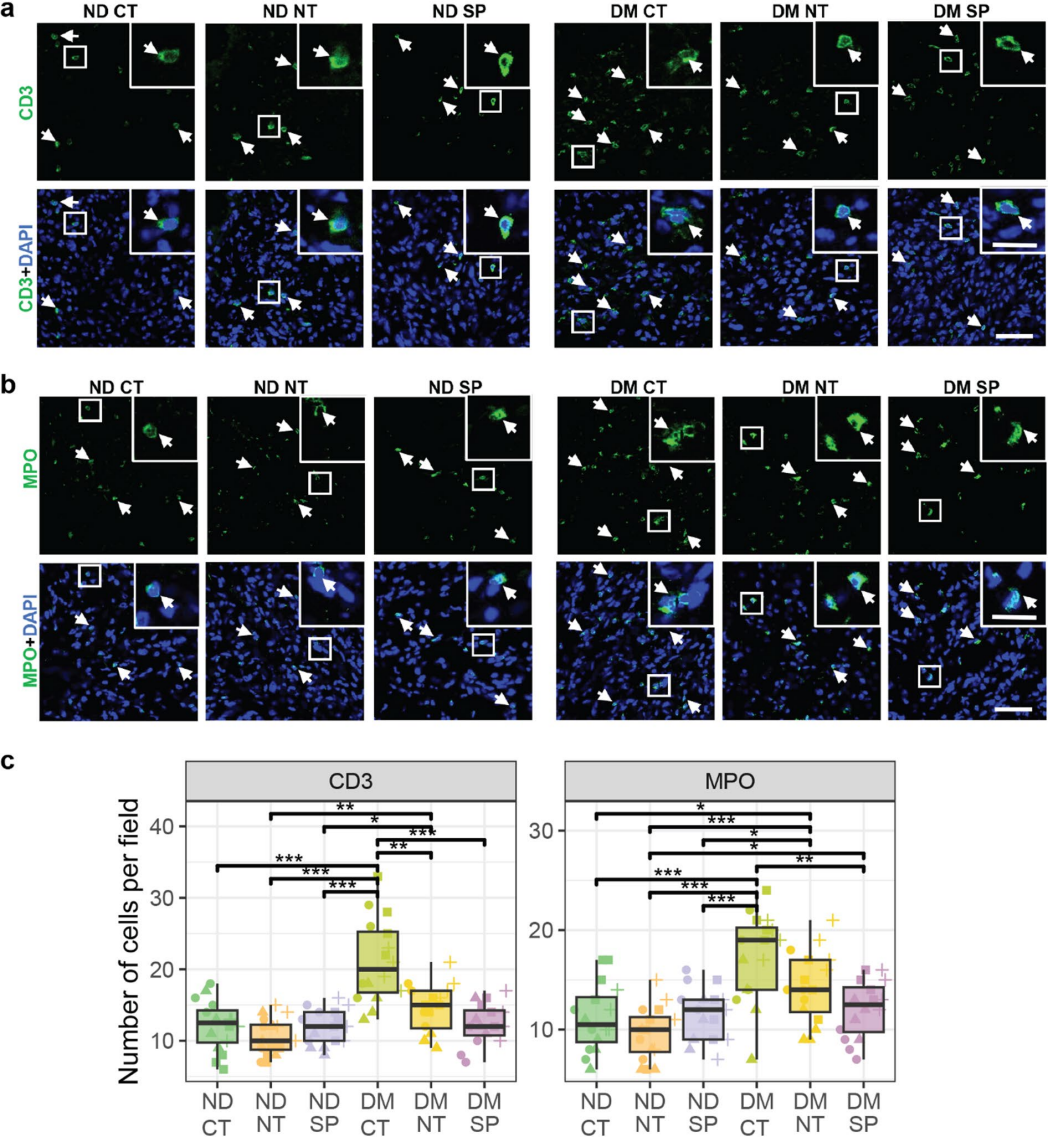
Neuropeptides modulate immune cell infiltration in diabetic wounds. (**A**) CD3 + cells and (**B**) neutrophils. T-cells were identified by CD3 (green) expression, and neutrophils were identified by myeloperoxidase (MPO, green). (**C**) Number of CD3 + cells and neutrophils per field at the wound site. The nuclei were stained with DAPI (blue). Scale bar − 50 μm; magnification: scale bar − 20 μm. Data are presented as mean ± SD from 3 biological replicates per group. White arrows identify the staining. Statistical significance was determined using one-way ANOVA with Welch correction and post hoc t-tests. *p < 0.05, **p < 0.01, ***p < 0.0001. Test statistics are reported in Table S2. ND- Non-diabetic; DM- Diabetic; CT – saline; NT-neurotensin; SP-Substance P.

To further assess the inflammatory response, we quantified neutrophils using MPO staining and T lymphocytes using CD3 marker. DM mice wounds had elevated numbers of CD3-positive cells (21.1 ± 6), which decreased with both NT and SP treatments (14.6 ± 3 and 12.4 ± 3, respectively) (Fig. 3A, C) to levels statistically comparable to CT ND mice (12.1 ± 4).

MPO-positive cells were elevated in DM mice wounds (17.4 ± 4), with both NT and SP treatment reducing this count (14.3 ± 4, 12.1 ± 3, respectively). NT treatment in DM mice remained significantly different than all ND groups. In contrast, SP-treated DM mice wounds (12.1 ± 3) exhibited only a slight, non-significant increase in MPO-positive cells compared to ND mice (11.2 ± 3) and a statistically different decrease from DM CT mice wounds (21.1 ± 6) (Fig. 3B, C, Table S2). These immunohistochemical analysis demonstrate that both NT and SP reduced inflammation in skin wounds, especially in diabetic skin wounds, with SP consistently producing more pronounced anti-inflammatory effects across macrophage polarization, lymphocyte infiltration, and neutrophil recruitment. These findings are consistent with the wound healing kinetics, indicating that diabetic wounds treated with neuropeptides heal faster.

### Baseline skin Microbiome composition in diabetic and non-diabetic mice

Skin swabs collected on day 0 from intact dorsal skin (Fig S2) revealed distinct microbial profiles between non-diabetic (ND) and diabetic (DM) mice. At the phylum level, ND mice showed a balanced distribution of Firmicutes (54.0%) and Actinobacteria (43.6%), whereas DM mice were overwhelmingly dominated by Firmicutes (91.1%). (Fig. S3).

At the genus level, ND mice were enriched in *Lactobacillus* (25.0%), followed by OTUs that clustered as Micrococcaceae_unclassified (21.6%), and Actinobacteria_unclassified (19.5%) (Fig. 3A). Among the *Lactobacillus* OTUs, Otu003 (12.8%), Otu005 (9.4%) and Otu011 (1.4%), contributed to its abundance (Fig. S4). *Staphylococcus* accounted for 13.3% of the average relative abundance in ND mice, detected only in 10 out of 12 mice. This was mainly driven by Otu001 (10.3%, 8/12), Otu007 (1.8%, 3/12), and Otu019 (1.0%, 3/12) (Fig. S4).

**Fig. 4:**
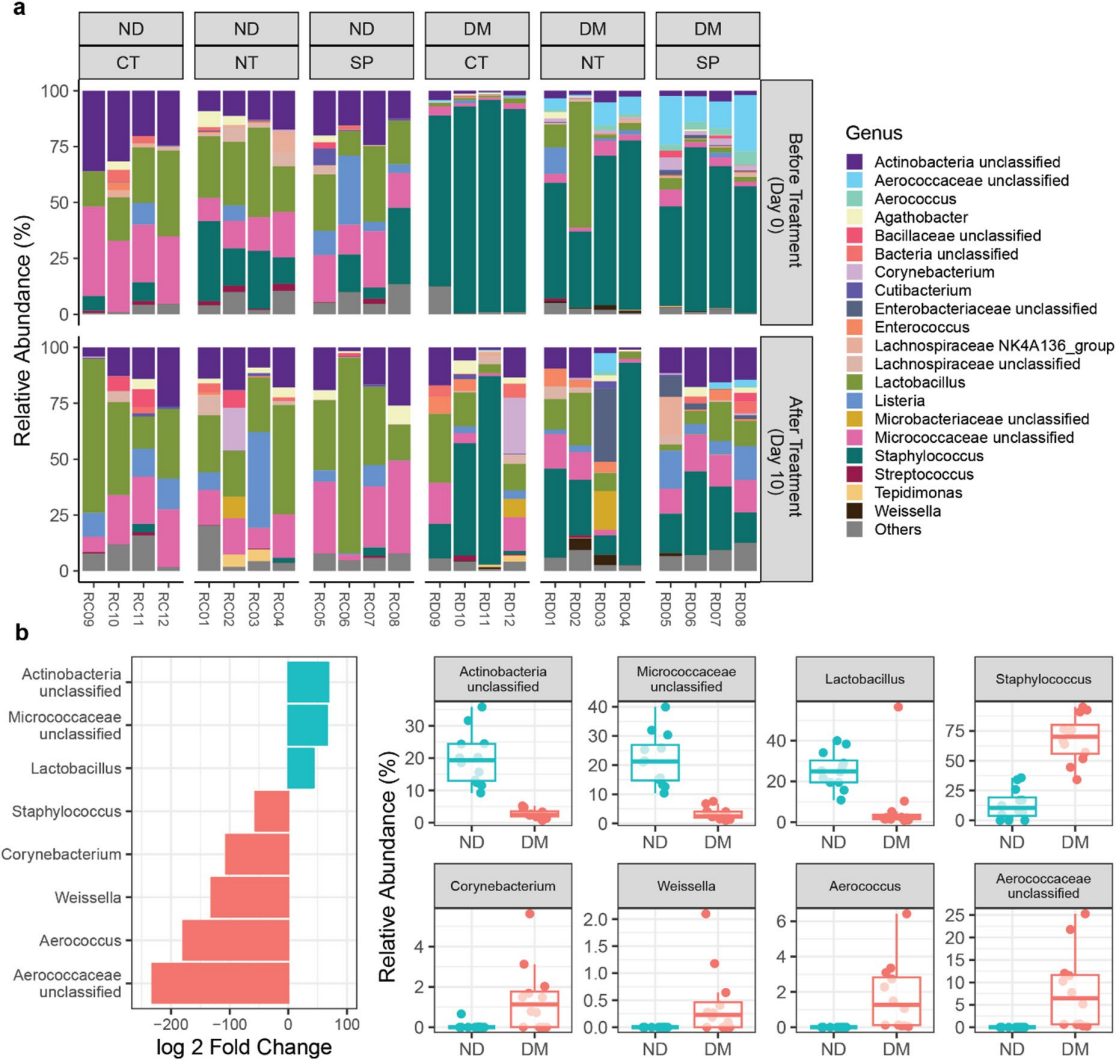
Diabetic (DM) mice display distinct skin microbiota compared to non-diabetic (ND) controls. (**A**) Relative abundance of the most prevalent and abundant genera detected in ND and DM mice, before and after treatment. Only genera present in at least 6 samples from either ND or DM mice were included. Colored boxes represent the 20 most abundant genera; grey boxes represent the 25 less abundant genera. (**B**) Differential abundance analysis of bacterial genera between ND and DM mice. Log₂ fold change was calculated as the log₂ ratio of mean relative abundance in ND versus DM mice. Positive values indicate genera enriched in ND mice; negative values indicate enrichment in DM mice. Test statistics are reported in Table S3.

Conversely, *Staphylococcus* dominated the skin bacteriome of DM mice (68.4%), largely due to Otu001 (67.1%), with minor contributions from Otu037 (0.5%), Otu108 (0.2%), and Otu019 (0.2%, 7/12) (Fig. S4). Other abundant genera consistently detected in DM mice included Aerococcaceae_unclassified (8.1%), *Lactobacillus* (7.3%), Micrococcaceae_unclassified (3.4%), Actinobacteria_unclassified (2.7%), *Aerococcus* (1.7%) and *Corynebacterium* (1.4%, present in 9/12 mice) (Fig. 3A). Notably, the detection of Aerococcaceae_unclassified, *Aerococcus* and *Weissela* (0.4%) was limited to DM mice (Fig. 4A).

Differential abundance analysis confirmed that Actinobacteria_unclassified, Micrococcaceae_unclassified, and *Lactobacillus* (particularly Otu003 and Otu005; Fig. S4) were enriched in ND mice (Fig. 3B, Table S3), while *Staphylococcus* (notably OTUs 108, 037, and 001; Fig. S5), *Corynebacterium*, *Weissella*, *Aerococcus*, and Aerococcaceae_unclassified were significantly higher in DM mice (Fig. 4B and S5, Table S3).

**Fig. 5:**
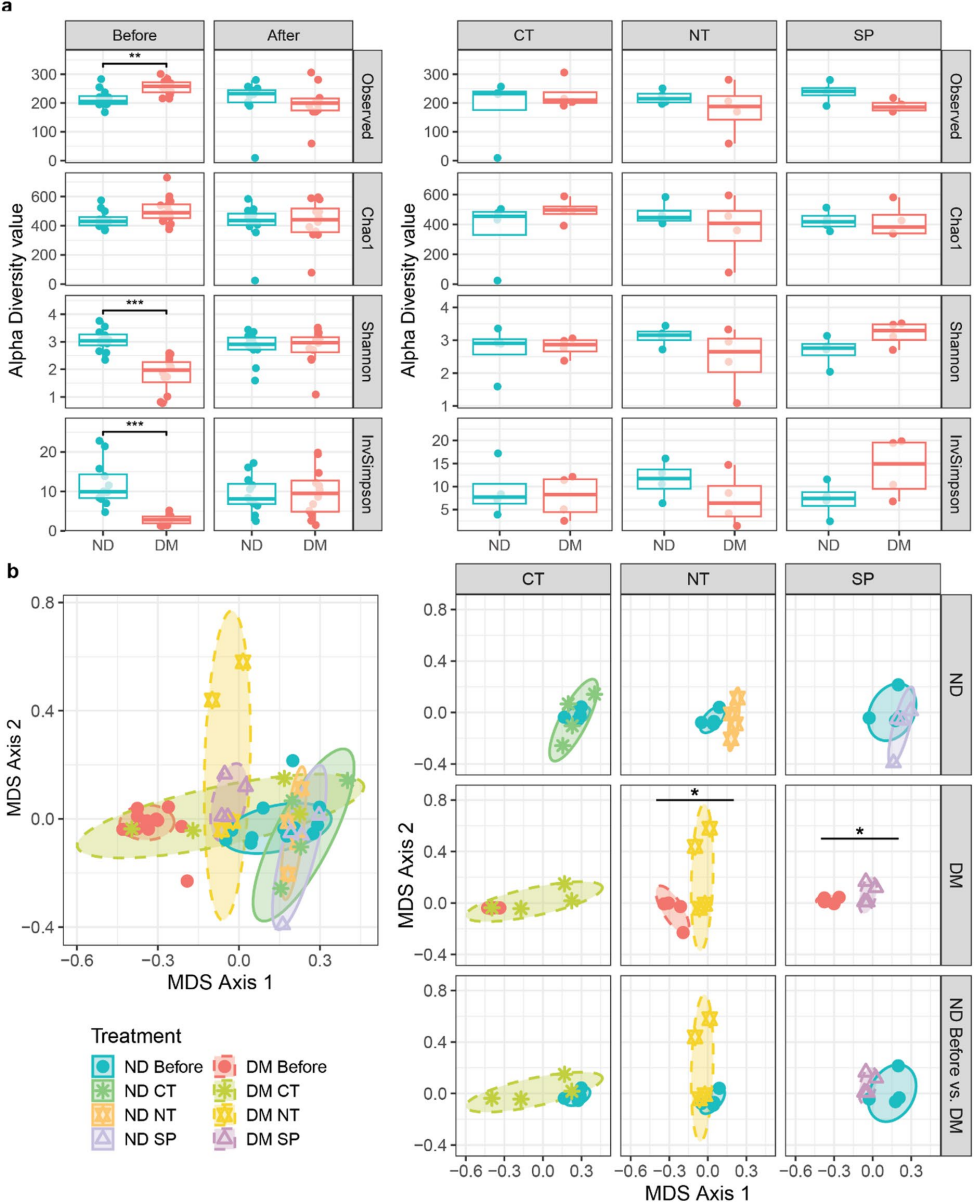
Substance P treatment partially restores microbial community structure in diabetic (DM) mice to resemble non-diabetic (ND) profiles. (**A**) Alpha diversity metrics of skin-associated bacterial communities in ND and DM mice before and after treatment with saline (CT), neurotensin (NT), or substance P (SP). Diversity was measured using Observed species, Chao1, Shannon, and Inverse Simpson indices (*n* = 24) Significant differences between groups were determined using Mann − Whitney − Wilcoxon Test with Halbach correction. *p < 0.05. Detailed statistics are provided in Table S4. (**B**) beta diversity analysis of the skin-associated bacterial communities among sampled mice using non-metric multidimensional scaling (NMDS) based on Bray–Curtis dissimilarity. Each point represents an individual mouse sample (*n* = 24). Colored symbols indicate treatment group; dashed lines distinguish DM status. PERMANOVA *p* = 0.0011; F = 5.3; R² = 0.48; NMDS stress = 0.138. Detailed pairwise comparisons are provided in Table S5.

Alpha diversity metrics revealed that DM mice harbored a greater number of unique OTUs (*p* = 0.00677, Fig. 5A, Table S4), while ND microbiota was more evenly distributed, as indicated by higher Shannon (*p* = 1.0 × 10^− 5^) and Inverse Simpson indices (*p* = 5.9 × 10^− 6^), suggesting a more balanced and resilient ecosystem.

Beta diversity analysis further confirmed distinct community structures between ND and DM mice (p − value = 0.0011; Fig. 5B, Table S5).

### Healing is associated with specific changes in the skin microbiota profile

To investigate treatment effects, wounds were treated daily with saline (CT), NT, SP. After 10 days, skin swabs from wound sites were profiled, regardless of closure status.

ND mice wounds largely retained their baseline structure, remaining dominated by *Lactobacillus* (33.7%), Micrococcaceae_unclassified (22.6%), and Actinobacteria_unclassified (15.8%). However, *Lactobacillus* prevalence decreased to 3 out of 4 mice in ND CT. *Staphylococcus* prevalence and abundance markedly decreased across treatments dropping to 0.82%. *Staphylococcus* was detected in only one NT treated and two CT mice. Interestingly, SP-treated wounds retained *Staphylococcus* in 3 of 4 mice, though abundance remained very low (0.96%), suggesting SP promoted a more structured microbial remodeling process. (Fig. 4A).

In DM mice, treatment markedly altered microbiota composition. Although *Staphylococcus* remained dominant, its abundance decreased by ~ 34%. Concurrently, *Lactobacillus*, Micrococcaceae_unclassified, and Actinobacteria_unclassified increased from 4.5% to a combined 11.0%. Aerococcaceae_unclassified and *Aerococcus*, found exclusively in DM mice, also declined following treatment (− 6.8%, −1.5%, respectively), with the largest decrease occurring in SP-treated DM mice. *Weissella*, likewise exclusively detected in DM mice, increased in CT and NT groups (+ 1.4%) however the small increase in SP (+ 0.05%, due to an increase of OTU 044 in one sample) (Fig. S3), was offset by *Weissella* becoming undetectable in the other 3 samples (Fig. 4A).

Treatment decreased the number of unique OTUs in DM mice and led to a more evenly distributed microbial community in diabetic mice, across all treatments (CT, NT and SP) (Fig. 5A). Although not statistically significant, SP-treated DM mice displayed the greatest improvement in evenness.

Beta diversity analysis revealed no significant differences in the bacterial community composition of ND mice before and after treatment (Fig. 5B, Table S5). In contrast, DM mice exhibited significant shifts in bacterial communities following NT and SP treatment conditions, more closely resembling ND controls than their own pre-treatment profiles (*p* = 0.0299, *p* = 0.0256) (Fig. 5B, Table S5). Interestingly, the bacterial community composition of DM CT mice was highly variable, resulting in some retaining baseline-like profiles (Fig. 5B).

**Fig. 6:**
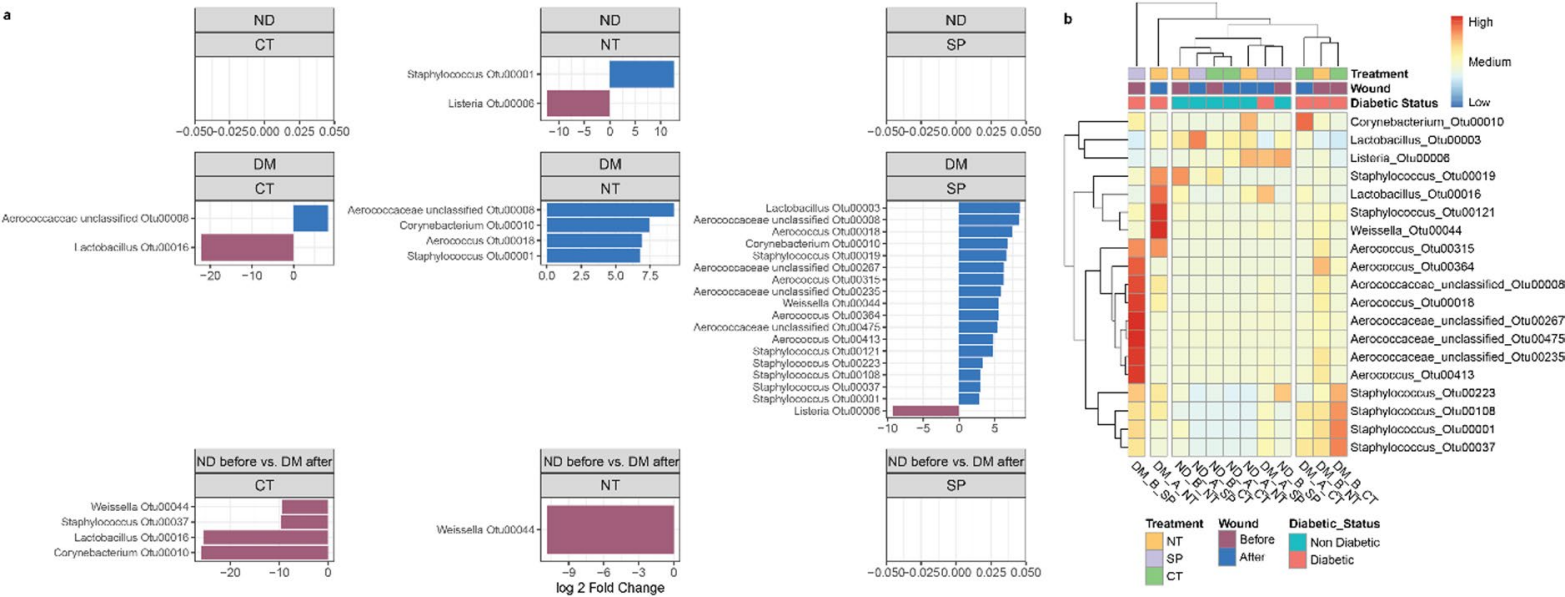
Substance P treatment in diabetic (DM) mice leads to the most pronounced shifts in OTU-level microbial composition. (**A**) Differential abundance analysis of operational taxonomic units (OTUs) in skin microbiota of DM mice before and after treatment with saline (CT), neurotensin (NT), or substance P (SP). Only OTUs showing significant changes by DESeq2 (*p* < 0.05) are shown. (**B**) Heatmap with hierarchical clustering based on OTU relative abundance profiles before and after treatment. Clustering reveals grouping patterns across treatment and DM status. Details and statistics are provided in Table S6.

Differential abundance analysis highlighted specific OTUs associated with the microbial remodeling occurring in DM mice. SP treatment was associated with reduction of *Staphylococcus* OTUs (including Otu001, Otu019, and Otu037, Otu108, Otu121 and Otu223), *Aerococcus* (Otu018, Otu315, Otu364, and Otu413) and Aerococcaceae_unclassified (Otu008, Otu235, Otu267 and Otu475) (Fig. 6A, Table S6). NT-treated DM mice showed similar but less extensive reductions, with significant changes limited to Aerococcaceae_unclassified Otu008, *Aerococcus* Otu018, and *Staphylococcus* Otu001 (Fig. 6A and S6). Importantly, SP-treated DM mice did not differ significantly from ND controls (Fig. 6A), suggesting that SP treatment shifts the skin microbiota towards a healthier, non-diabetic-like profile, consistent with clustering analyses (Figs. 5B and 6B). CT mice largely clustered with pre-treatment profiles, reflecting highly variable post-treatment dynamics, whereas NT-treated mice formed a separate cluster, driven by greater inter-sample variability in key OTUs such as Aerococcaceae_unclassified Otu008, *Aerococcus* Otu018, and *Staphylococcus* Otu019 (Fig. 6B). For example, in DM CT mice, *Staphylococcus* OTUs (Otu001, Otu037, Otu180) remained highly variable after treatment, with Otu037 the only significantly enriched relative to ND controls (Fig. 6), despite *Staphylococcus* abundance being highly enriched in the pre-treatment samples. Similarly, Aerococcaceae_unclassified Otu008 remained significantly associated with the pre-treatment state, despite the low pre-treatment amount of Aerococcus or Aerococcaceae_unclassified among all the DM mice., limiting the observable impact of treatment on these OTUs.

Additional patterns supported these findings, *Corynebacterium* Otu010, elevated in DM mice at baseline, decreased after both NT (-0.93%) and SP (-2.7%) treatments but increased in CT wounds (+ 6.3%) (Fig. 6), suggesting further untreated DM wounds fail to restore microbial balance. Similarly, *Weissella* Otu044, absent in ND mice, increased in CT and NT groups but was largely eliminated after SP treatment (Fig. 6).

## Discussion

Chronic wounds in diabetic patients remain a major clinical challenge, driven by persistent inflammation, vascular dysfunction, hyperglycemia, and a dysbiotic skin microbiota. Hyperglycemia promotes pro-inflammatory cytokine release and macrophage polarization toward the M1 phenotype, suppressing pro-healing M2 macrophages and perpetuating a vicious cycle of unresolved inflammation^20^. Dysbiotic microbial communities with high bacterial burden and biofilm formation further delay healing^18^.

In this study, we demonstrate that topical application of neurotensin (NT) or substance P (SP) significantly accelerates wound healing in diabetic mice. Whereas saline-treated (CT) diabetic wounds required 9.1 days to achieve 50% closure, NT and SP reduced this time to 7.5 and 7.3 days, respectively. These findings align with prior work demonstrating the regenerative role of neuropeptides^29,30^ and highlight their therapeutic potential in overcoming impaired diabetic wound repair.

Diabetic mice presented a higher number of pro-inflammatory M1 macrophages compared to non-diabetic controls, consistent with previous observations of persistent inflammation in diabetic wounds^29,36^. Notably, wounds treated with either NT or SP showed a significant reduction in M1 macrophages and an increase in M2 macrophages, reflecting a transition to a pro-healing inflammatory state. The SP-treated wounds exhibited a macrophage polarization profile (M1/M2 ratio) indistinguishable from non-diabetic controls, underscoring SP’s effectiveness in resolving chronic inflammation and promoting normal wound healing. Further, both neuropeptides reduced infiltration of CD3 + T cells and MPO + neutrophils to the wound site. These immune cells are typically elevated in chronic wounds and contribute to tissue damage and fibrosis^37,38^. The ability of SP to bring these markers to levels similar to, or even lower, than those observed in non-diabetic mice further highlights its higher immunomodulatory capacity.

Skin microbiome analysis revealed that Actinobacteria and Firmicutes were the dominant bacterial phyla across all mice, consistent with their role as the primary constituents of the skin microbiota^9^. Non-diabetic mice exhibited a microbiome dominated by *Lactobacillus*, Micrococcaceae_unclassified, Actinobacteria_unclassified, and *Staphylococcus.* These commensals play specific roles in wound healing, such as modulating the inflammatory response and promoting normal inflammation levels. *Lactobacillus* species, often overlooked due to their low relative abundance in human skin microbiome, have documented immunomodulatory properties, including the ability to induce M2 macrophage differentiation^39,40^. In contrast, diabetic mice displayed a skin microbiome heavily skewed towards *Staphylococcus*, and harbored distinct taxa, including Aerococcaceae_unclassified, *Aerococcus*, and *Weissella*. These microbial profiles align with previous findings on diabetic skin dysbiosis, characterized by an overrepresentation of *Staphylococcus*, *Aerococcus*, and Proteobacteria and a reduction of *Streptococcus* and *Lachnospiraceae*^41^. Importantly, these shifts, along with the presence of *Weissella*, have previously been implicated in delayed healing in diabetic wounds^13,25,42^.

Prior to treatment, diabetic mice showed higher microbial richness, primarily driven by the presence of rare, low-abundance taxa with uneven distribution. This pattern is consistent with previous findings showing that chronic wounds often exhibit microbial instability and reduced ecological evenness^41^, mirroring dysbiosis observed in human diabetic skin^42,43^. The wound healing process resulted in a significant reduction in the number of unique OTUs in diabetic mice, with microbial communities becoming more evenly distributed. While the skin microbiota of non-diabetic mice remained fairly stable before and after treatment, diabetic wounds exhibited significant microbial restructuring following treatment. All treatments, including saline, altered the microbial community composition to some extent, however, SP-treated wounds displayed the most pronounced shift. Notably, there were sharp reductions in *Staphylococcus* (− 33.8%), Aerococcaceae_unclassified (− 6.9%) and *Aerococcus* (− 1.5%). Additionally, less abundant genus such as *Weissella*, that disappeared in three of the four SP-treated samples, and *Corynebacterium* (−2.7%) with 50% reduction in prevalence after SP-treatment, were significantly diminished. These findings support the idea that preventing the establishment of stable pathogenic microbial communities enhances healing^25^. Although chronic wound microbiomes are inherently dynamic, persistence of stable pathogenic profiles is often associated with poor outcomes. Opportunistic pathogens of genera such as *Staphylococcus* spp., *Pseudomonas* spp., and *Corynebacterium* spp. commonly dominate diabetic foot ulcers, contributing to delayed healing through biofilm formation and secretion of virulence factors. The presence of specific *Staphylococcus* strains and strict or facultative anaerobes like *Aerococcus* is linked to delayed healing and poor clinical outcomes^23,24,26,44^. Therefore, restoring microbial balance, especially when facilitated by immunomodulatory agents like SP, appears critical for re-establishing cutaneous homeostasis.

Our findings align with prior evidence showing that diabetic skin undergoes selective microbial shifts including bacteria such as *Staphylococcus*, *Aerococcus*, and *Weissella*, changes that are closely intertwined with the skin’s response to injury^44^. Importantly, preventing the establishment of an abnormal, stable microbial community in wounds has been shown to promote faster healing^25^. Furthermore, numerous studies have shown that wounding itself can significantly reshape the cutaneous microbiome, often enabling opportunistic pathogens to exploit newly available ecological niches. This microbial disruption often leads to a reduction in bacterial diversity in chronic wounds^43^, as well as in acute traumatic fracture wounds^45,46^. In addition, in acute injuries, the microbiome of wounded tissue initially diverges from that of adjacent healthy skin, but eventually the two converge over time as healing progresses^45,46^. This pattern suggests a dynamic and restorative role of the skin microbiome in wound resolution, one that is particularly evident when healing is not hindered by diabetes-related impairments.

Our results further support that this microbial remodeling is closely intertwined with immune responses, as evidenced by the observed changes in macrophage polarization and other immune cells. SP’s ability to modulate both immune and microbial dynamics appears central to its superior efficacy in promoting wound healing. Taken together, these results reinforce the concept of the skin microbiota as an active participant in wound healing rather than a passive bystander. The bidirectional interactions between commensals microbes and the host’s immune system, including the role of microbial metabolites and microbial-immune crosstalk, are essential for regulating inflammation, maintaining skin barrier integrity and promoting tissue repair^12,21^.

Although NT and SP demonstrated marked effects on wound closure, inflammatory cell infiltration, and microbial community structure, several considerations are important when assessing their translational potential. First, while neuropeptides have entered clinical testing in other therapeutic areas, including neurotensin receptor–targeted radioligands for oncology (ClinicalTrials.gov NCT03525392), neurotensin-loaded silk fibroin membranes for oral wound repair (ClinicalTrials.gov NCT05191082), and substance P delivery for recent-onset type 1 diabetes (ClinicalTrials.gov NCT02820558), among others, no NT- or SP-based agonist therapies have yet been evaluated clinically for cutaneous wound healing. Advancing these candidates toward clinical translation will therefore require essential preclinical steps, including formulation optimization to improve peptide stability, dose–response studies, good laboratory practices (GLP)-compliant toxicology and biodistribution analyses, and development of delivery systems compatible with regulatory standards^47^. Human pilot studies assessing safety and preliminary efficacy in diabetic wounds would then be necessary to establish clinical feasibility.

This study demonstrates that topical treatment with the neuropeptides SP and NT act at the interface of the nervous, immune and microbial systems to restore the balance disrupted in diabetic wounds. They accelerate wound healing in diabetic mice by promoting immune resolution, shifting macrophage polarization toward a pro-healing (M2) phenotype, and restructuring the skin microbiome toward a more balanced and beneficial composition.

This study also has several limitations. The murine excisional wound model, while widely used, does not fully replicate the structural, vascular, or microbial complexity of human diabetic foot ulcers, and therefore cannot capture all features of chronic human wound pathophysiology. Microbiome sampling was limited to two time points, restricting temporal resolution and limiting our ability to infer causal relationships between microbial shifts, inflammatory modulation, and wound closure. The relatively small cohort size, particularly for 16 S rRNA sequencing, may have reduced sensitivity to detect low-abundance taxa or strain-level differences with functional significance. We further note that wound area measurements reflect the combined effects of contraction and re-epithelialization. Because murine wounds can heal partially through contraction, and neuropeptides can also have an influence in contraction, future studies using splinted wound models, together with histological quantification of myofibroblast activation (α-SMA), will be needed to dissect the relative contributions of contraction versus keratinocyte migration to the accelerated closure observed with NT and SP. Moreover, the absence of early post-injury timepoints prevents us from determining whether NT or SP also influence the initiation of acute inflammation, a mechanism previously described for SP in diabetic wounds^29^. Since the inflammatory condition of the wounds were performed at day 10 post-injury, we can only assess effects on persistent inflammation and its resolution rather than the immediate acute response. Thus, while our findings support a role for NT and SP in correcting persistent inflammatory imbalance and promoting resolution, we cannot exclude additional effects on early-phase activation. Future studies with high-temporal-resolution sampling in the first days after injury will be required to clarify whether NT/SP modulate early acute responses, accelerate the transition to resolution, or act through both mechanisms. Additional work on long-term safety, formulation stability, and scalable manufacturing will also be necessary to support clinical translation of NT or SP.

These considerations highlight both the promise of neuropeptide-based approaches and the need for rigorous, stepwise translational studies. High-resolution longitudinal analyses, improved microbiome sampling strategies, functional assays, and clinically viable delivery platforms will be essential to bridge the gap between the present preclinical findings and future therapeutic application in chronic diabetic wounds.

The findings presented here highlight the potential of neuropeptide-based therapies, particularly through early intervention to prevent the establishment of a dysbiotic microbial community and foster a microenvironment conducive to healing. These findings suggest that neuropeptide-based therapies hold promise to improve wound resolution while reducing reliance on conventional antimicrobials, which is particularly valuable in the face of rising antimicrobial resistance and the challenges posed by biofilm-associated treatment failures.

## Methods

### Animals

C57BL/6J mice (Charles River Laboratories, France), 8-week-old male, were housed in certified local facilities under standard conditions (12-hour light/dark cycle, normal room temperature) with *ad libitum* access to food and water. All the experimental protocols involving animals were approved by the animal research ethics committee of the Center for Neuroscience and Cell Biology and the Faculty of Medicine of the University of Coimbra (ORBEA_213_2019/28082019) and by the national (Directorate-General for Food and Veterinary of the Portuguese Ministry of Agriculture) research ethical committee. Also, the animal protocols were in accordance with the European Directive 2010/63/EU and the Portuguese Decree-law (113/2013) for the use of animals for scientific purposes. This study is also reported in accordance with ARRIVE guidelines (https://arriveguidelines.org).

### Diabetes induction

Diabetes was induced by daily intraperitoneal injections of streptozotocin (STZ, 50 mg/kg) for five consecutive days. After one week, mice with blood glucose levels above 250 mg/dL were considered diabetic. The animals were kept diabetic for 6 weeks prior to the wound healing experiment. Weight and glycemia were measured prior to the wound healing experiment (Fig. S1). The animals were grouped in cages of 4.

### Animal model of wound healing

Dorsal hair was removed 4 days before wounding, and the baseline (day 0) skin microbiota was collected by swabbing. Mice were individually housed and given analgesia (buprenorphine, 0.05 mg/kg subcutaneously before wounding; 0.1 mg/kg every 6–8 h for up to 24 h after). Under isoflurane anesthesia (5% induction, 2.5% maintenance in 0.5 L/min O₂), skin was sterilized with betadine and two 6-mm full-thickness wounds created using a punch biopsy tool.

Animals were assigned to six groups (*n* = 4/group): ND (to which the vehicle saline was applied), ND + NT (50 µg/wound), ND + SP (32 µg/wound), DM (saline), DM + NT (50 µg/wound), and DM + SP (32 µg/wound). Substance P and neurotensin were obtained from Bachem AG, Bubendorf, Switzerland. Treatments were administered topically once daily to the wounds. Wound size was photographed every 2 days and quantified with Fiji (v2.14.0/1.54f, NIH Image, USA).

On day 10 after wounding, mice were anesthetized with ketamine/xylazine (100/10 mg/kg, intraperitoneally) and euthanized by cervical dislocation. Microbiota swabs were again collected. All swabs (day 0 and 10) were preserved at − 80 °C. Wounded skin was harvested and cryopreserved in optimal cutting temperature gel (OCT, VWR) at − 80 °C for immunohistochemistry.

### Immunohistochemistry

The presence of M1 and M2 macrophages (CD68 + TNF-α and CD68 + CD206 respectively), T lymphocytes (CD3), and neutrophils (myeloperoxidase, MPO) present in the wounded skin was accessed by immunohistochemistry^36^.

Skin cryosections (10 μm thickness) were fixed in ice-cold acetone for 10 min, and permeabilized at RT with PBS with 1% tween (PBS-T) and 0.2% Triton X-100 for 30 min. Subsequently, the samples were blocked with 50 µl of 10% goat serum for 30 min at RT. Then, the samples were placed in a humidified chamber and incubated overnight at 4 °C with the primary antibodies: rabbit anti-CD68 (1:100, Abcam, UK) and rat anti-TNF-α (1:200, AbD Serotec, Portugal) for M1 macrophages; rat anti-CD206 (1:200, Santa Cruz, Santa Cruz, USA) for M2 macrophages; rabbit anti-CD3 (1:100, Abcam, UK) for T-cells; rabbit anti-MPO (1:100, Abcam, UK) for neutrophils. The samples were then incubated at room temperature (RT) for 1 h, with DAPI (1:1000) for nuclei staining and the secondary antibody, anti-rat (1:500, conjugated to Alexa Fluor 568, Invitrogen) and anti-rabbit (1:500, Alexa Fluor 488 conjugated, Invitrogen). The 3–5 random images at the wound site were obtained using the Carl Zeiss LMS 710 confocal microscope, with 400x magnification, and acquired using the Zen Blue software. The number of cells was analyzed using Fiji software (v2.14.0/1.54f, NIH Image, USA).

### Microbiome profiling by 16 S rRNA gene sequencing

Skin swabs stored at − 80°C (< 15 days) were processed by Eurofins Genomics for microbial DNA extraction and sequencing on the Illumina MiSeq^®^ platform (Illumina, USA). The V4–V5 regions of the 16S rRNA gene were amplified using universal primers 515F-Y (5’- GTGYCAGCMGCCGCGGTAA-3’) and reverse primer 926R (5’-CCGYCAATTYMTTTRAGTTT-3’). Raw data processing, sequence clustering, and taxonomic annotation were performed in mothur (v1.44.1, www.mothur.org) using the SILVA reference database (release 138).

### Statistical analysis

All analyses were performed in R (v4.0.4). Data wrangling and visualization used tidyverse (v1.3.0)^48^. Wound size and immune cell counts were compared by one-way ANOVA with Welch’s correction, followed by post hoc pairwise t-tests. Linear regression (stats::lm, squared-transformed data) was applied to assess relationships between wound size and healing time. Significance was set at *p* < 0.05.

Microbiota analyses were performed with phyloseq (v1.42.0), microbiome (v1.20.0), and vegan (v2.6-4)^49–51^ packages in R. Alpha diversity (phyloseq::estimate_richness) was tested by Mann–Whitney–Wilcoxon with Benjamini–Hochberg correction. Low-prevalence OTUs (< 6 observations across all samples) and those absent from at least one treatment group (< 3 observations) were removed. OTU abundances were standardized to the median sequencing depth. Beta diversity was calculated with Bray–Curtis dissimilarity (vegan::vegdist) and visualized by NMDS; group differences were assessed by PERMANOVA (adonis2, 9999 permutations)^52^, accounting for matched samples.

Differential abundance was assessed at genus and OTU level using the Mann–Whitney–Wilcoxon test (BH correction), ALDEx2 t-test, DESeq2 Wald test, and PERMANOVA^52–54^. For untreated ND vs. DM comparisons, taxa were considered significant if at least two tests were in agreement. For before/after treatment comparisons, only the DESeq2 Wald test was applied due to sample size. Heatmaps were generated with pheatmap (v1.0.12).

## Supplementary Information

Below is the link to the electronic supplementary material.

Supplementary Material 1

Supplementary Material 2

## Data Availability

The DNA sequencing raw data have been submitted to the BioProject collection of the National Center for Biotechnology, with accession number PRJNA1338077. The datasets analyzed during the current study are available from the corresponding author upon reasonable request.
