# Supplementary material for: Neuropeptides neurotensin and substance P accelerate diabetic wound healing by modulating immunity and the skin microbiome
Source: Sci Rep. 2026 Feb 17;16:9456. doi: 10.1038/s41598-025-30723-w (PMC13004966; doi:10.1038/s41598-025-30723-w)
Supplement: Supplementary file 1 — Supplementary Material 1 [file 41598_2025_30723_MOESM1_ESM.pdf]

SUPLEMENTAL INFORMATION FILE

Neuropeptides Neurotensin and Substance P accelerate Diabetic Wound Healing by Modulating Immunity and the Skin Microbiome

Ana Maranha#* [1,2,] Ermelindo C. Leal#* [1,2] , Susana Alarico [1,2,] Igor Tiago [3,4], Sónia G. Pereira [5], Nuno Empadinhas [1,2], Eugénia Carvalho [1,2]

1 CNC—Center for Neuroscience and Cell Biology, University of Coimbra, 3004-504 Coimbra, Portugal
2 CIBB—Centre for Innovative Biomedicine and Biotechnology, University of Coimbra, 3004-504 Coimbra, Portugal
3 Department of Life Sciences, University of Coimbra, 3000-456 Coimbra, Portugal
4 CFE – Centre for Functional Ecology, University of Coimbra, 3000-456 Coimbra, Portugal
5 ciTechCare – Center for Innovative Care and Health Technology, School of Health Sciences (ESSLei), Polytechnic University of Leiria, 2414-016 Leiria, Portugal

# These authors contributed equally
* Correspondence should be addressed to ana.maranha@cnc.uc.pt or to ecleal@cnc.uc.pt

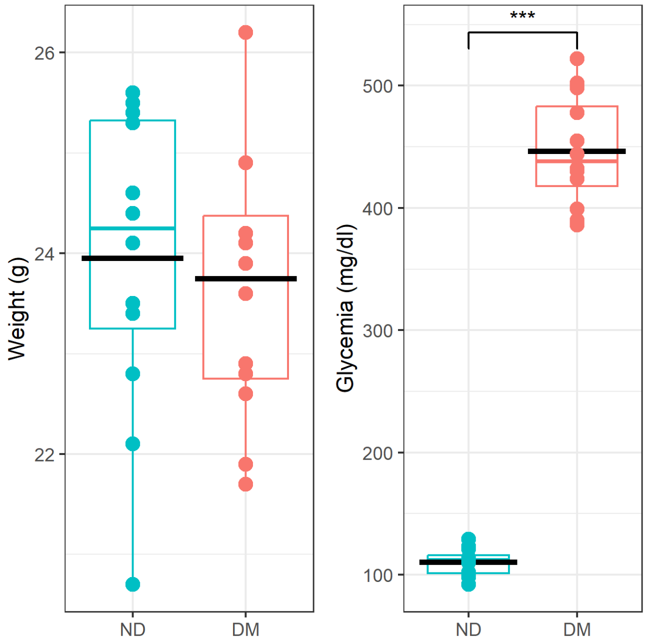

Fig. S1 Weight and blood glucose levels measured in non-diabetic (ND) and diabetes mellitus (DM) mice after 8 weeks of diabetes induction, before the wound healing experiment. Black crossbar represents means. $*p<0.05$, $**p<0.01$, $***p<0.0001$

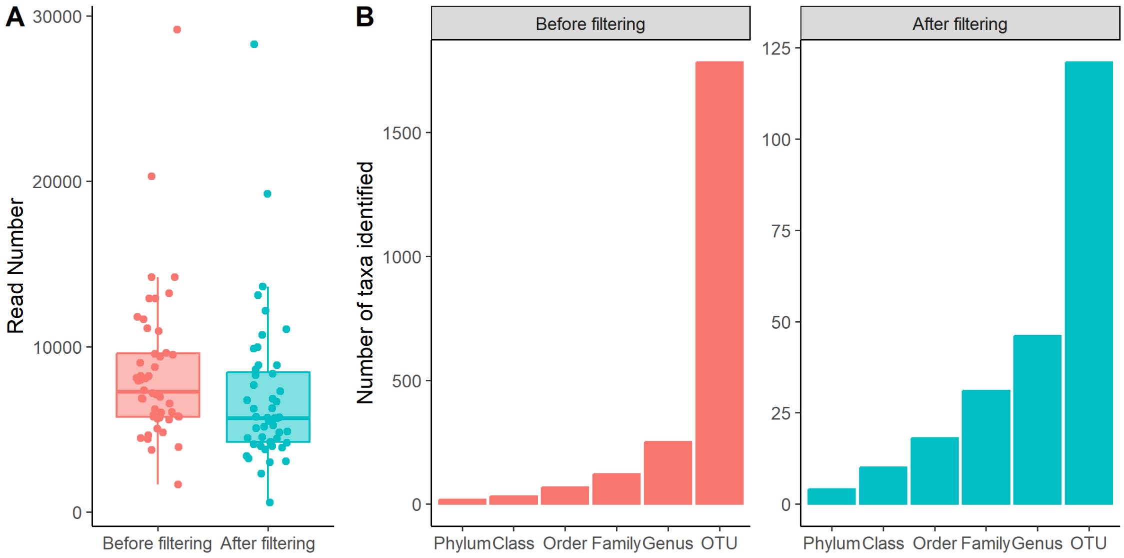

Fig. S2 Sequencing yield and number of taxa identified. A) Across all samples, sequencing yielded an average of 8476.5±4569.7 reads per sample B) identifying a total of 1,784 OTUs, which grouped into 251 genera, 120 families, 67 orders, 32 classes, and 17 bacterial phyla. For downstream analyses, only the most prevalent taxa (present in ≥6 samples) were included, resulting in 121 OTUs clustering into 45 genera across, 31 families, 18 orders, 10 classes, 4 phyla.

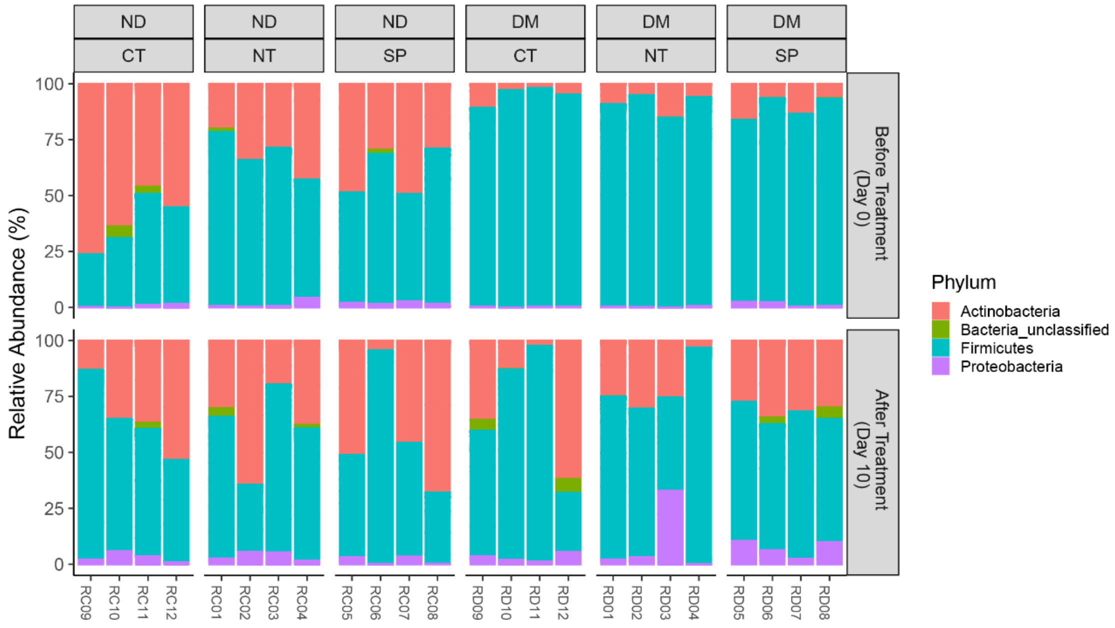

Fig. S3 Phylum level clustering of the most abundant and prevalent genus on the skin of sampled mice shown in Fig. 3. Phyla detected in non-diabetic (ND) and diabetic (DM) mice, before and after treatment for each treatment group (CT, NT, SP).

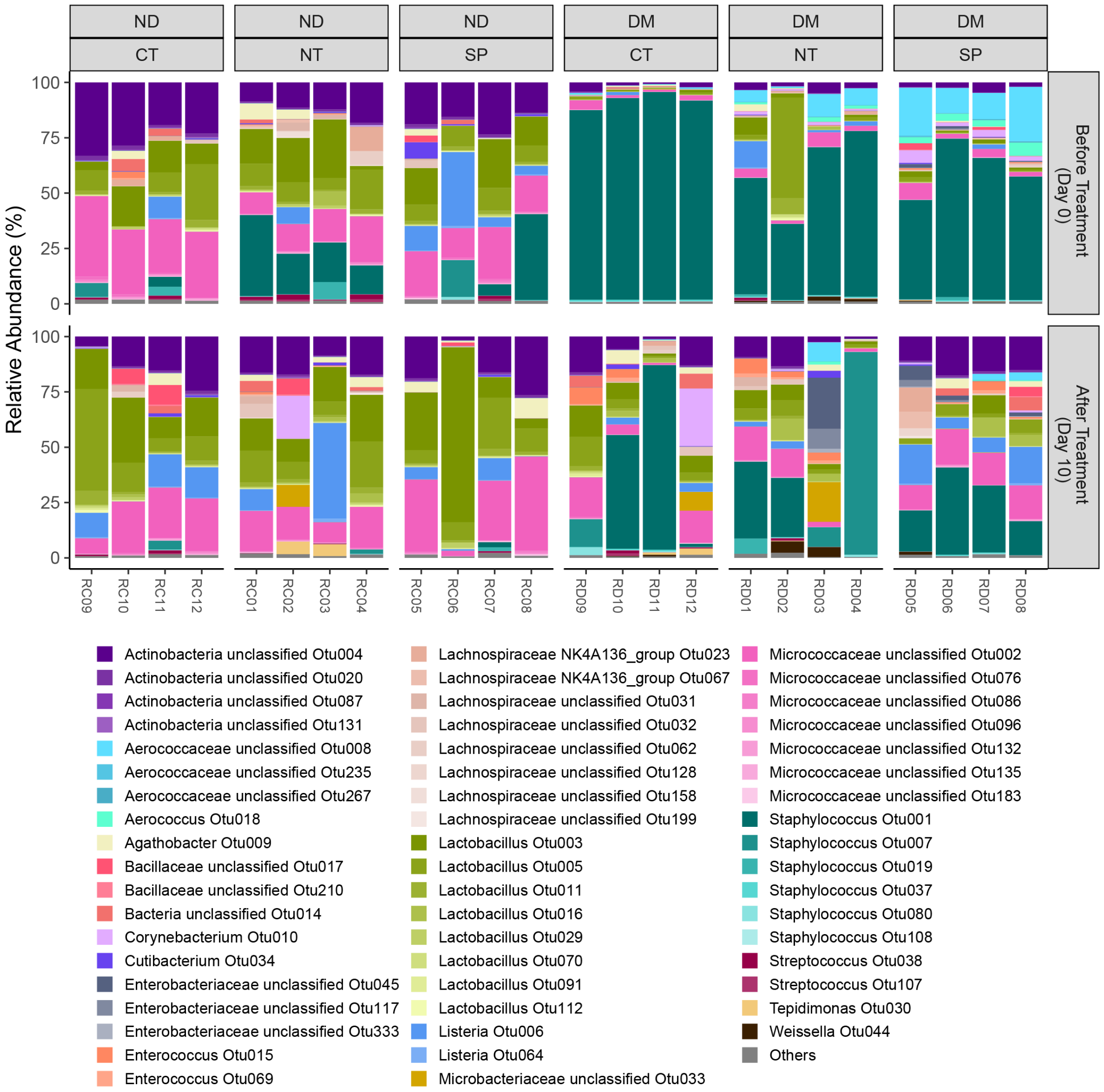

Fig. S4 OTU composition of the most abundant and prevalent genus on the skin of sampled mice shown in Fig. 3. Colored boxes specify the 55 most abundant OTUs. Grey box represents the 28 less abundant OTUs. OTUs detected in non-diabetic (ND) and diabetic (DM) mice, before and after treatment for each treatment group (CT, NT, SP).

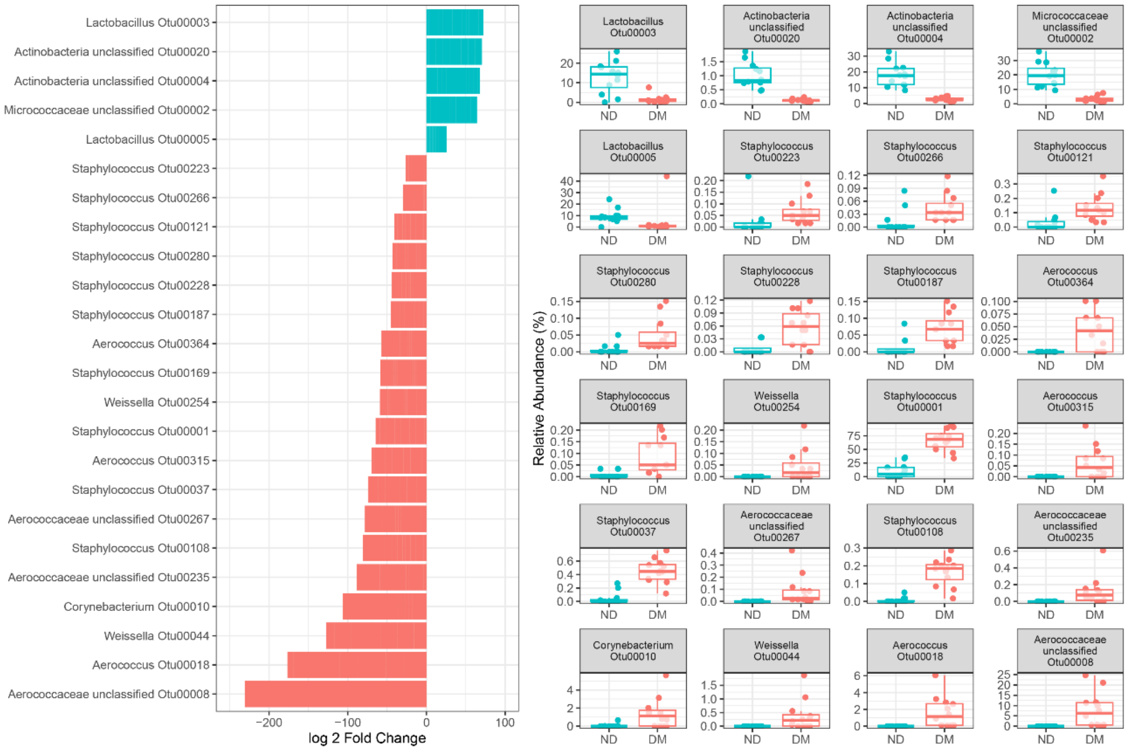

Fig. S5 Differential abundance of OTUs present in non-diabetic (ND) vs. diabetic (DM) mice before treatment. Blue represents ND mice and red represents DM mice.

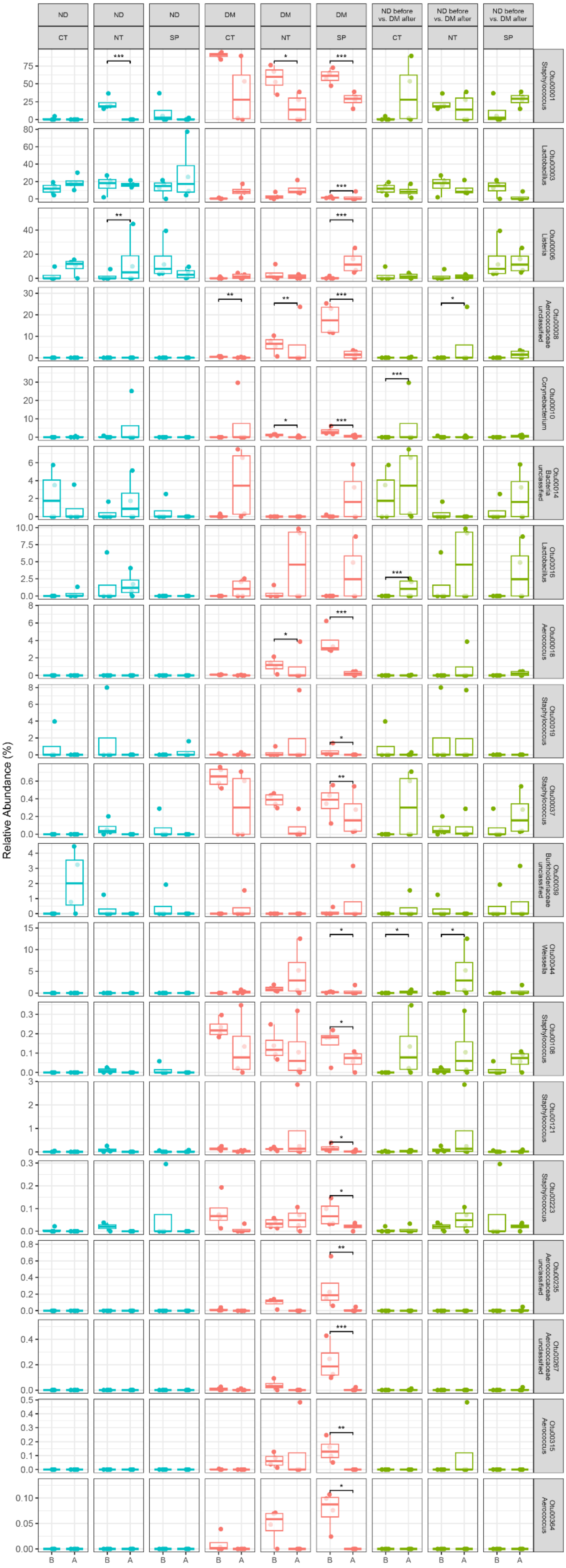

ND
CT
NT
SP
DM
ND before vs. DM after
Otu0001 Staphylococcus
Otu0003 Lactobacillus
Otu0006 Listeria
Otu0008 Aerococcaceae unclassified
Otu00010 Corynebacterium
Otu00014 Bacteria unclassified
Otu00016 Lactobacillus
Otu00018 Aerococcus
Otu00019 Staphylococcus
Otu00037 Staphylococcus
Otu00039 Burkholderiaceae unclassified
Otu00044 Weissella
Otu00108 Staphylococcus
Otu00121 Staphylococcus
Otu00223 Staphylococcus
Otu00235 Aerococcaceae unclassified
Otu00267 Aerococcaceae unclassified
Otu00315 Aerococcus
Otu00364 Aerococcus
Relative Abundance (%)
B
A

Fig. S6 Differentially abundant OTUs in non-diabetic (ND) and diabetic (DM) mice after treatment (denoted as "A") compared to their respective pre-treatment states (denoted as "B"). Additionally, comparisons are shown between post-treatment DM mice and pre-treatment ND mice (used the baseline healthy microbiota), for each treatment group (CT, NT, SP). $^*p < 0.05$, $^{**}p < 0.01$, $^{***}p < 0.0001$.
